# Supplementary material for: Evaluation of the antidiarrheal activity of dichloromethane-methanol crude extract of the aerial parts of Croton kinondoensis (Euphorbiaceae) in Mice
Source: PLoS One. 2025 Oct 3;20(10):e0333527. doi: 10.1371/journal.pone.0333527 (PMC12494273; doi:10.1371/journal.pone.0333527)
Supplement: Attachment — Submitted filename: Response to Reviewers.docx [file pone.0333527.s004.docx]

**Response to Reviewers:**

We sincerely thank the reviewers for their thoughtful and constructive feedback on our manuscript, Evaluation of the Antidiarrheal Activity of Dichloromethane-Methanol Crude Extract of the Aerial Parts of *Croton kinondoensis* (Euphorbiaceae) in Mice. We appreciate the time and effort the reviewers and editors have dedicated to evaluating our work. Their insightful comments have helped us to improve the clarity, rigor, and overall quality of the paper.

In the following, we provide a detailed, point by point response to each of the reviewers’ comments. For ease of reference, reviewer comments are included in italics, followed by our responses and a summary of the corresponding revisions made to the manuscript.

We hope that the revisions address the concerns raised and that the improved manuscript meets the expectations of the reviewers and editors.

### ****Reviewer #1 Comment:****

*The topic presented in the abstract is contemporary, novel, pertinent, and impactful.*

**Response:**
Thank you for your positive feedback. We are pleased that you found the topic contemporary and impactful. We believe our study addresses a significant gap by scientifically validating a traditionally used plant for managing diarrhea.

### ****Reviewer #1 Comment:****

*The backdrop is skillfully established in the introduction, and the goals are in line with the field's current priorities.*

**Response:**
We appreciate your recognition of our introduction and alignment with current research priorities. We aimed to highlight both the global burden of diarrhea and the urgent need for alternative therapies such as plant-based remedies.

### ****Reviewer #1 Comment:****

*I urge the writer/writers to be more explicit in their technique with greater details...*

**Response:**
Thank you for this valuable suggestion. In response, we have revised the **Methods** section to provide more clarity and detail on:

- Animal grouping and dosing: Specified five groups (n = 6) and exact doses (100, 200, 400 mg/kg).
- Definitions of outcome measures: Clearly stated how antidiarrheal activity and gastrointestinal motility were measured.
- Experimental timeline: Included timing for extract administration, castor oil challenge, charcoal meal test, and observation intervals.

### ****Reviewer #1 Comment:****

*...and provide succinct, specific results to back up the conclusion...*

**Response:**
We have revised the **Abstract** and **Results** sections to include specific numerical data summarizing key findings. For instance:

- Antidiarrheal inhibition: “400 mg/kg inhibited diarrhea by 25.57%, compared to 71.43% by loperamide.”
- Antimotility effect: “400 mg/kg of the extract reduced intestinal transit by 27.98%, compared to 33.43% by loperamide.”

### ****Reviewer #1 Comment:****

...to improve the proposal. This is a promising effort all around, however, it might need a few minor edits to make it more comprehensive and clearer.

**Response:**
We appreciate your encouragement. In addition to refining the abstract and methods, we also polished the language throughout the manuscript for clarity and coherence. We ensured that the conclusions are well-supported by the data and clearly linked to the study objectives.

### ****Reviewer #2 Comment 1:****

“If you were utilized to conduct the experiment using mice, it is obvious that your experiment is in vivo. So, I suggest the authors to remove in vivo under your title.”

**Response:**
Thank you for the observation. We agree with the suggestion and have revised the title to remove the phrase “In Vivo,” as the use of mice clearly implies an in vivo study.

**Revised Title:**

**“Evaluation of the Antidiarrheal Activity of Dichloromethane-Methanol Crude Extract of the Aerial Parts of Croton kinondoensis (Euphorbiaceae) in Mice”**

### ****Reviewer #2 Comment 2:****

“Please modify the affiliation and make it complete. Try to mention the list of the authors and their respective affiliation. You have mentioned the study is conduct at United States International University but it is incomplete and include the country (Kenya)”

**Response:**
Thank you for pointing this out. We have updated the authorship and affiliation section to clearly list all authors with their respective institutional details, including the country.

### ****Reviewer #2 Comment 3:****

“Regarding the keywords, I recommended to modify it since unnecessary words are included which are not listed under the title like medicinal plants, phytochemicals, plant extracts, gastrointestinal motility,,,,,,,etc. In sum, you have missed the main words. Just try to look the title then you can find the key words.”

**Response:**
Thank you for your constructive feedback. We have revised the keyword list to align more closely with the core concepts reflected in the title. The updated keywords now focus on the key elements of the study including the plant species, methodology, and pharmacological effect.

**Revised Keywords:**

Croton kinondoensis, antidiarrheal activity, dichloromethane-methanol extract, Swiss albino mice, Euphorbiaceae, castor oil-induced diarrhea

### ****Reviewer #2 Comment 4:****

“Why you use “dichloromethane-Methanol” as extracting solvent. What makes it preferable from other solvents like methanol, ethanol and then like?”

**Response:**
Thank you for this important question. We have added a justification for the use of the dichloromethane-methanol (1:1) solvent system in the **Plant Material Collection and Extraction** subsection of the Methodology.

**Inserted Justification in Manuscript:**

“The 1:1 dichloromethane-methanol (DCM:MeOH) solvent system was selected to facilitate the extraction of both polar and moderately non-polar phytoconstituents. Dichloromethane aids in solubilizing less polar compounds such as terpenoids and steroids, while methanol efficiently extracts polar compounds including flavonoids, alkaloids, and phenolics. This mixed solvent approach has been shown to enhance the yield and diversity of secondary metabolites from medicinal plants, particularly when screening for broad pharmacological activity such as antidiarrheal effects (Abubakar & Haque, 2020).”

### We have also added the following to the list of References:

Abubakar AR, Haque M. Preparation of Medicinal Plants: Basic Extraction and Fractionation Procedures for Experimental Purposes. J Pharm Bioallied Sci. 2020;12(1):1–10. <https://www.ncbi.nlm.nih.gov/pmc/articles/PMC7398001/>

### ****Reviewer #2 Comment 5:****

“In the abstract section, your findings are not evidenced with P values. So, please include the p values in each metrics to show the level of significance.”

**Response:**
Thank you for the observation. We have revised the abstract to include p-values for the key metrics, reflecting statistical significance in the main findings.

**Revised Segment in Abstract (Results section):**

“In the castor oil-induced diarrhea model, the extract at 400 mg/kg inhibited diarrhea by 25.57% (p < 0.05), compared to 71.43% inhibition by loperamide (p < 0.001). In the charcoal meal test, 400 mg/kg of the extract reduced intestinal transit by 27.98% (p < 0.01), compared to 33.43% by loperamide (p < 0.001).”

### ****Reviewer #2 Comment 6:****

“Why not the authors conduct castor oil-induced enteropooling test which is the pillar test to evaluate the antidiarrheal activity?”

**Response:**
Thank you for this suggestion. While we acknowledge the importance of the castor oil-induced enteropooling test as a classical method for evaluating antisecretory effects, this study focused on the **motility** and **frequency of defecation** components of diarrhea using:

- **Castor oil-induced diarrhea test,** and
- **Charcoal meal transit test**

We aimed to characterize both the **delay in defecation onset** and **inhibition of motility**, which are relevant to the traditional claims about C. kinondoensis. We recognize the enteropooling model would add value and recommend it for future mechanistic studies in follow-up research.

**We have added the following in the Discussion section:**

“Although the castor oil-induced enteropooling test is a standard model for assessing intestinal fluid secretion, it was not included in this study. Future investigations will incorporate this method to evaluate potential antisecretory mechanisms of C. kinondoensis extract.”

### ****Reviewer #2 Comment 7:****

“Why not the authors present the findings which align with the method section. The authors tried to assess the onset of defecation and the total fecal output but this metrics are absent in the result section.”

**Response:**
Thank you for identifying this gap. We have updated the Results section to include:

- **Onset of diarrhea (time to first wet stool)**
- **Total fecal output (wet + dry feces)**

These results have now been included in both narrative and tabular form for consistency with the stated methods.

**We have added the following to the Results section:**

“The onset of diarrhea was delayed in a dose-dependent manner by the extract. At 400 mg/kg, the onset was delayed by 42.3 minutes compared to 19.7 minutes in the negative control (p < 0.05). Total fecal output was also reduced significantly at higher doses of the extract compared to the control group.”

**Reviewer #3 Comment 1:**

*“As a general comment, there are some grammatical topographical errors.”*

**Response:**
Thank you for this observation. We have carefully reviewed the entire manuscript for grammatical, typographical, and stylistic errors. We corrected minor language issues, including punctuation, word choice, and sentence clarity to improve the overall readability and professionalism of the manuscript.

**Reviewer #3 Comment 2:**

*“In the abstract section, specifically under the conclusion, based on the finding you have concluded that the plant has moderate antidiarrheal activity. I am confused since you need to have a standard to say mild, moderate and strong antidiarrheal activity. Please reason out for this conclusion.”*

**Response:**
Thank you for raising this important point. We agree that any qualitative classification (e.g., mild, moderate, strong) should be based on a defined and referenced standard. To address this, we have clarified in the **Methodology section** that our interpretation of the antidiarrheal effect was based on previously established criteria used in published studies (Degu et al., 2016; Sisay et al., 2017). Specifically, inhibition of diarrhea:

- Less than 20% is considered **mild**,
- Between 20–40% is **moderate**,
- Between 40–60% is **strong**, and
- Greater than 60% is considered **very strong**.

Based on these criteria, the 25.57% inhibition observed at the 400 mg/kg dose of C. kinondoensis falls within the **moderate** range. This explanation has been included in both the **Methods** and **Conclusion** sections of the manuscript to provide transparency and justify our terminology.

**We have also added the following to the list of References:**

Degu A, Engidawork E, Shibeshi W. Evaluation of the anti-diarrheal activity of the leaf extract of *Croton macrostachyus* in mice model. *BMC Complement Altern Med*. 2016;16:379.

Sisay M, Engidawork E, Shibeshi W. Evaluation of the antidiarrheal activity of the leaf extracts of *Myrtus communis Linn*. *BMC Complement Altern Med*. 2017;17(1):103.

### ****Reviewer #3 Comment 3:****

“In the conclusion part of the abstract section, you have written as ‘While its efficacy was lower than loperamide, the extract demonstrated significant potential as a natural remedy for diarrhea.’ Please omit ‘while’ and replace with ‘although’.”

**Response:**
Thank you for this language improvement suggestion. We have revised the sentence in the abstract conclusion as recommended for better grammatical structure and flow.

**Revised sentence in the Abstract (Conclusion section):**

“Although its efficacy was lower than loperamide, the extract demonstrated significant potential as a natural remedy for diarrhea.”

### ****Reviewer #3 Comment 4:****

“The authors tried to conduct phytochemical screening tests for 5 secondary metabolites. Why you left the rest like steroids, glycosides? At least it is important to include steroids under your phytochemical test because it has so many pharmacological activities.”

**Response:**
Thank you for the valuable suggestion. We appreciate the importance of broad phytochemical screening. In fact, additional tests for **steroids and glycosides** were conducted and are now included in the revised manuscript. The methodology section under **Phytochemical Screening** and **Table 1** has been updated.

### ****Reviewer #3 Comment 5:****

Under your method section, particularly on acute toxicity study, there is a paragraph which seems the result for acute toxicity test but not appropriate place. Rather include under the result section. “The results indicated that no mortality or severe toxicity signs were observed...” So, I suggest removing from the method section.

**Response:**
Thank you for the observation. We agree that outcome statements should be reported in the Results section. Accordingly, the sentence “The results indicated that no mortality or severe toxicity signs were observed...” has been removed from the Methods section and appropriately repositioned under the **Results** section describing the acute toxicity findings.

### ****Reviewer #3 Comment 6:****

In castor oil-induced diarrheal model, the authors plan to assess the overall antidiarrheal activity, but I saw from this model that you have planned to assess the “general behavior of the mice”. So, please omit it from this subsection. Rather include it under acute toxicity if necessary. Also, I saw a statement at the end of the 4-hour observation period, mice were carefully examined for signs of dehydration, discomfort, or toxicity. Please remove. Your plan is only to assess its overall antidiarrheal activity, not observing and assessing the toxicity.

**Response:**
Thank you for pointing this out. We have revised the **Castor Oil-Induced Diarrhea Model** subsection by removing the statements related to "general behavior of the mice" and “dehydration or discomfort.”

### ****Reviewer #3 – Comment 7:****

Under the method section, in both models, the authors tried to explain how castor oil induces diarrhea and intestinal motility. Please remove this statement as it is not necessary to narrate about its effect; rather include under discussion section.

**Response:**
We appreciate this observation. The explanatory details about how castor oil induces diarrhea and stimulates intestinal motility have been removed from the **Methods** section and will instead be appropriately discussed under the **Discussion** section to support interpretation of the experimental results.

### ****Reviewer #3 – Comment 8:****

In the method section, to evaluate the antidiarrheal activity, you used only 2 models. Why didn’t the authors include the third model (castor oil-induced enteropooling test)? This is mandatory and the most pillar model, particularly to check the extract's effect on intestinal fluid accumulation.

**Response:**
Thank you for highlighting this important point. We acknowledge the significance of the castor oil-induced enteropooling test in evaluating fluid accumulation, a key aspect of antidiarrheal action. Due to limitations in resources and time, this model was not included in the current study. However, we recognize its value and have noted it as a **limitation** of the study in the Discussion section, with a recommendation for inclusion in future investigations to fully assess the extract’s antidiarrheal mechanisms.

### ****Reviewer #3 – Comment 9:****

Under the result section, regarding phytochemical screening test, please don’t repeat the method. The authors have already mentioned how they conducted the phytochemical test. Hence, please narrate only the findings.

**Response:**
Thank you for your insightful feedback. In response, we have revised the **Results** section on phytochemical screening to focus solely on the observed findings. All methodological descriptions have been removed from this section, as they are already detailed under **Materials and Methods**. The revised text now presents a concise summary of the detected and undetected phytochemical classes and their potential pharmacological relevance, as appropriate for a results-focused presentation.

### ****Reviewer #3 – Comment 10:****

In the method section, the authors didn’t plan to conduct phytochemical tests for saponins, but I have seen from the result section that the test is negative for saponins. Similarly for steroids, glycosides, anthraquinones, and reducing sugars. Why?

**Response:**
Thank you for your critical observation. Initially, the manuscript did not explicitly include the tests for saponins, steroids, glycosides, anthraquinones, and reducing sugars in the **Methods** section. We acknowledge this oversight and have now revised the Methods section to clearly include the phytochemical screening tests performed for these additional compounds. This correction ensures consistency between the methodology and the results reported.

### ****Reviewer #3 – Comment 11:****

Similarly, as comment 13, in the result section, you repeat the methods. Therefore, please omit it since the authors have already written under the method section. This is my comment for acute toxicity test, castor oil induced diarrheal model, and intestinal motility test, and then like.

**Response:**
We appreciate your feedback. As suggested, we have removed all redundant methodological descriptions from the **Results** section, including those related to the acute toxicity test, castor oil-induced diarrheal model, and gastrointestinal motility model. The Results section now focuses exclusively on outcome data, while procedural details remain solely under the **Materials and Methods** section.

### ****Reviewer #3 – Comment 12:****

Under the result section of castor oil-induced diarrheal model, the authors tried to show findings but not in accordance to the method. The authors were planning to assess the onset of diarrhea and total fecal output. However, I haven’t seen the results for these metrics under the result section. Why? Please include them, since these parameters are significant when planning to assess the antidiarrheal activity.

**Response:**
Thank you for bringing this to our attention. We agree that reporting the **onset of diarrhea** and **total fecal output** is essential for a comprehensive assessment of antidiarrheal activity. These data were collected but were previously omitted from the Results section in error. We have now added the relevant findings and updated **Table 2** to include these metrics. This revision ensures alignment between the stated methodology and the presented results.

### ****Reviewer #3 – Comment 13:****

Here in the result section, you tried to discuss about the findings. Please only present the findings. The discussion is in the discussion part. Again, the authors are written about the onset of diarrhea and total fecal output but this metrics are absent under table 2. Why?

**Response:**
Thank you for this important comment. We have revised the **Results** section to strictly present the findings without any interpretation or discussion, moving all explanatory content to the **Discussion** section. Additionally, we have updated **Table 2** to include the **onset of diarrhea** and **total fecal output**, which were previously omitted due to formatting oversight. These parameters are now reported in alignment with the methodology and are important indicators of antidiarrheal activity.

### ****Reviewer #3 – Comment 14:****

In table 3, the investigators include % inhibition of diarrhea. Please replace with % inhibition of peristaltic index (PI).

**Response:**
We appreciate your observation. As suggested, **Table 3** has been corrected to replace “% inhibition of diarrhea” with **“% inhibition of peristaltic index (PI)”.**

### ****Reviewer #3 – Comment 15:****

In the results of antimotility test, you tried to discuss about the findings. Please only present the findings.

**Response:**
Thank you for pointing this out. We have revised the **antimotility test results** section to present the findings objectively without interpretation. All analytical and comparative statements have been moved to the **Discussion** section to maintain clarity and structural accuracy.

### ****Reviewer #3 – Comment 16:****

In the discussion section, please cite the references. I have seen some blank statements which need evidence. For instance, “Diarrhea remains a significant,,,,,,,,and pharmaceutical treatments is often limited” but not cited.

**Response:**
Thank you for noting the missing citations. We have carefully reviewed the **Discussion** section and inserted appropriate references to support key claims.

### ****Reviewer #3 – Comment 17:****

In the discussion section, you have also discussed about the onset of diarrhea and total fecal output which are absent under your result section. Why you discussed on this metrics?

**Response:**
Thank you for your observation. We acknowledge that the **onset of diarrhea** and **total fecal output** were initially missing from the **Results** section. These metrics were indeed part of the data collected and are essential to the interpretation of antidiarrheal activity. We have now **updated the Results section and Table 2** to include these metrics. The discussion on these parameters is now appropriately supported by their inclusion in the results.

### ****Reviewer #3 – Comment 18:****

In this section, the references are not cited at the proper place. Eg. Additionally, (31) reported that,,,,,,,. Please rewrite and cite at the end of the statement.

**Response:**
Thank you for identifying this citation formatting issue. We have carefully reviewed the entire **Discussion section** and corrected all in-text citations to ensure they follow proper scientific writing standards.

### ****Reviewer #3 – Comment 19:****

Under discussion section, as a general comment some statements or paragraphs are not cited. Please cite them.

**Response:**
We appreciate your attention to detail. A thorough review of the **Discussion section** has been conducted, and **relevant citations have now been added.**

### ****Reviewer #4 – Comment 1:****

No rationale for the extraction using 1: DCM-MEOH.

**Response:**
Thank you for this observation. The choice of **dichloromethane: methanol (1:1)** as the extraction solvent was based on its **ability to extract a wide polarity range of phytochemicals**, including both moderately polar and non-polar compounds, which may contribute to the plant’s antidiarrheal activity. This solvent combination is widely reported in ethnopharmacological studies for effectively yielding bioactive secondary metabolites such as alkaloids, flavonoids, and terpenoids. A rationale for this choice has now been included in the revised **Materials and Methods** section with appropriate citation.

### ****Reviewer #4 – Comment 2:****

The effective dose of the plant extract used in the study is physiologically irrelevant.

**Response:**
We acknowledge your concern regarding the extract doses. The selected doses (100, 200, and 400 mg/kg) were **based on traditional usage** and **previous preclinical studies** assessing safety and efficacy of crude extracts in rodent models. Although high doses may be necessary in crude extract studies due to the complex mixture of compounds and low concentration of active constituents, they serve as a **preliminary screening tool** for identifying potential pharmacological activity. These findings can guide future dose-refinement and bioassay-guided fractionation studies to isolate and test purified compounds at lower, physiologically relevant doses.

### ****Reviewer #4 – Comment 3:****

The traditional phytochemical screening is extremely outdated. Almost all of aerial parts of medicinal plants contain to some extent flavonoids/other phenolics, phytosteroids, and sugars. What’s new?

**Response:**
We understand and appreciate your concern. The **preliminary phytochemical screening** serves as an **initial step** in characterizing the crude extract's general chemical profile, consistent with standard practice in ethnopharmacological validation studies. While many aerial parts of plants contain these classes of compounds, the value of this study lies in **linking the presence of these constituents with observed biological activity** in a species that is **locally used for treating diarrhea but remains underreported in pharmacological literature.** We have emphasized this point in the revised **Discussion** section and noted the need for further studies using advanced techniques like GC-MS, LC-MS, or NMR to characterize specific bioactive constituents.

### ****Reviewer #4 – Comment 4:****

Any plant extract at 400 mg/kg can do the same effect!

**Response:**
We respectfully disagree with this generalization. While many plant extracts may show non-specific effects at high doses, the **effectiveness of Croton kinondoensis at 400 mg/kg was statistically significant compared to the negative control,** and consistent with the pharmacological profile of known antidiarrheal constituents such as flavonoids and tannins. Moreover, **dose-dependent trends were observed**, indicating a biological response rather than a non-specific irritant or placebo effect. Nonetheless, we agree that future work should involve **bioassay-guided fractionation** to isolate and test specific constituents at lower, standardized doses to enhance physiological relevance.
