# Supplementary material for: Evaluation of the antidiarrheal activity of dichloromethane-methanol crude extract of the aerial parts of Croton kinondoensis (Euphorbiaceae) in Mice
Source: PLoS One. 2025 Oct 3;20(10):e0333527. doi: 10.1371/journal.pone.0333527 (PMC12494273; doi:10.1371/journal.pone.0333527)
Supplement: Attachment — Submitted filename: Reviewer comments.docx [file pone.0333527.s002.docx]

**Date: 5/10/2025**

**Reviewer response letter to Academic Editor**

**Journal: PLOS ONE**

I thank you for your invitation to review this study entitled as “In Vivo  Evaluation of the Antidiarrheal Activity of Dichloromethane-Methanol Crude Extract of the Aerial Parts of Croton kinondoensis (Euphorbiaceae) in Mice”

General comments to authors. Improve language use

**Specific comments to author/authors**

1. If you were utilized to conduct the experiment using mice, it is obvious that your experiment is in vivo. So, I suggest the authors to remove in vivo under your title
2. Please modify the affiliation and make it complete. Try to mention the list of the authors and their respective affiliation. You have mentioned the study is conduct at United States International University but it is incomplete and include the country (Kenya)
3. Regarding the keywords, I recommended to modify it since unnecessary words are included which are not listed under the title like medicinal plants, phytochemicals, plant extracts, gastrointestinal motility,,,,,,,etc. in sum, you have missed the main words. Just try to look the title then you can find the key words.
4. Why you use “dichloromethane-Methanol” as extracting solvent. What makes it preferable from other solvents like methanol, ethanol and then like?
5. In the abstract section, your findings are not evidenced with P values. So, please include the p values in each metrics to show the level of significance
6. Why not the authors conduct castor oil induced enteropooling test which is the pillar test to evaluate the antidiarrheal activity?
7. Why not the authors present the findings which align with the method section. The authors tried to assess the onset of defecation and the total fecal output but this metrics are absent in the result section
